# Quantitative Analysis of Gender Stereotypes and Information Aggregation in a National Election

**DOI:** 10.1371/journal.pone.0058910

**Published:** 2013-03-26

**Authors:** Michele Tumminello, Salvatore Miccichè, Jan Varho, Jyrki Piilo, Rosario N. Mantegna

**Affiliations:** 1 Dipartimento di Scienze Statistiche e Matematiche “Silvio Vianelli”, University of Palermo, Palermo, Italy; 2 Department of Social and Decision Sciences, Carnegie Mellon University, Pittsburgh, Pennsylvania, United States of America; 3 Dipartimento di Fisica e Chimica, University of Palermo, Palermo, Italy; 4 Department of Physics and Astronomy, University of Turku, Turku, Finland; 5 Center for Network Science and Department of Economics, Central European University, Budapest, Hungary; Cinvestav-Merida, Mexico

## Abstract

By analyzing a database of a questionnaire answered by a large majority of candidates and elected in a parliamentary election, we quantitatively verify that (i) female candidates on average present political profiles which are more compassionate and more concerned with social welfare issues than male candidates and (ii) the voting procedure acts as a process of information aggregation. Our results show that information aggregation proceeds with at least two distinct paths. In the first case candidates characterize themselves with a political profile aiming to describe the profile of the majority of voters. This is typically the case of candidates of political parties which are competing for the center of the various political dimensions. In the second case, candidates choose a political profile manifesting a clear difference from opposite political profiles endorsed by candidates of a political party positioned at the opposite extreme of some political dimension.

## Introduction

Two widely investigated topics of political science concern the role of gender stereotypes of female and male candidates in elections [1]–[4] and the process of information aggregation occurring in a voting procedure [5], [6]. The process of information aggregation in a voting procedure has mainly been investigated so far in settings inspired by the earliest mathematical models of voting in elections dating back to Condorcet’s work in the eighteenth century. Specifically, recent works have focused on the information aggregation process occurring in a Jury or a Committee [7], [8] and on voting procedures where candidates are assumed to choose political profiles in response to information obtained by election results and exogenous factors leading to polarization in candidates’ choices [9], [10]. A line of research across economics and political science considers information aggregation in voting as an example where a social institutional infrastructure synthesizes information held by many people. The other prominent example of institution synthesizing information held by many people is the financial market where individual beliefs of investors are aggregated when they collectively act to perform price discovery [11].

The role of gender stereotypes has been mainly investigated to assess whether citizens utilize gender information to infer candidates’ political orientations, especially in the presence of low-informed voters [1]–[4]. Almost all studies found in the literature have been performed with empirical analyses investigating US and Canadian elections. The general consensus is that there are voters’ expectations of greater female competency on welfare issues, such as dealing with poverty or the aged, and less competency on military and defense issues [1]. In the framework of US politics, empirical evidence has been obtained showing that the greatest political distance in US House race is perceived by voters when opponent candidates are a Democratic female candidate against a Republican male candidate [2]. This observation suggests that women Democratic candidates have, or are perceived with, a political profile which is more liberal than the one of the corresponding male Democratic candidate.

In the present study, we investigate the role of gender stereotypes and information aggregation in a parliamentary election of a European country. Specifically, we investigate the election of the Finland’s parliament of April 2011. We choose to investigate this election because it is available detailed public information about the political profile publicly presented by a large set of candidates and elected. This is possible due to the initiative of Helsingin Sanomat, which is the largest Finnish newspaper. During the electoral campaign, Helsingin Sanomat asked all candidates to complete a questionnaire of 31 questions concerning welfare, pensions, the economy, taxes, defense, foreign and domestic affairs, municipalities and the central government. After the elections the database of the answers of all candidates was made available for research by Helsingin Sanomat.

By studying this special database, we are able to analyze the political profile made public by many candidates and elected. Specifically, we analyze the relationships between political profile and a series of information about the candidate comprising gender, party membership, electoral success (estimated by the number of obtained votes) and other metadata information. It should be noted that, differently from previous studies, our investigation is primarily focused on the political profile offered by candidates rather than on the ones perceived by informed or low-informed voters. In other words, we study the role of gender and party membership in the characterization of the political profile made public by candidates. We verify the presence of gender stereotypes in female candidates and we observe that successful candidates are in general more homogeneous in their political profile than non-elected candidates. We interpret this finding as a form of information aggregation realized by the voting procedure. We notice that the information aggregation follows at least two different patterns. In the first pattern, the most successful candidates are the ones able to make a political offer which summarizes the political expectations of the political center of the country. In the second pattern, successful political profiles manifest clear dissimilarity from opposite political parties that are representing political views which are different in policy positions and ideologies.

## Materials and Methods

We investigate the answers that candidates for the Finnish parliamentary elections held in April 2011 provided to a survey organized by Helsingin Sanomat. The purpose of the questionnaire was to allow voters to compare their views to those of the candidates made available online during the electoral campaign before the election, so as to make a more informed voting decision. A similar questionnaire by Helsingin Sanomat has been available for all elections since 2000, but here we focus only on the responses of the candidates for the 2011 parliamentary elections because the entire database of answers has been made available for research only for this election. Data is available and discussed in the online blog http://blogit.hs.fi/hsnext/.

The questionnaire has 31 questions (see supporting information file Questions S1). For 29 questions only one answer can be provided choosing it among a variable number of possible answers. In our analysis we use these 29 questions, leaving out two multiple-choice questions with 26 and 15 possible answers (Q21 and Q31 of the questionnaire). Among candidates, 1,803 out of a total number of 2,315 candidates answered the survey. Among them there are 181 out of the 200 parliament members eventually elected. In addition to all answers provided by the candidates, the data made available by Helsingin Sanomat also contains information on the respondents, including name, age, gender, party, election district and education level.

The 200 elected members of the Finnish parliament are chosen in 15 election districts. With the exception of the Åland district, which always elects a single member, the number of elected members of each district depends on the size of the electorate of the district. The election system is party-list proportional, using the D’Hondt method for allocating seats. Within parties, seats are allocated in the order of votes received.

In the 2011 elections 8 out of 17 political parties that nominated candidates obtained at least one seat in the parliament. In addition, one independent candidate was elected. Before the election there have been three large political parties, KESK (Center party, a centrist, agrarian, and liberal political party), KOK (National Coalition Party, a right-wing, pro-European political party), and SDP (the Social Democratic Party), but in the election PS (the True Finns, a populist and nationalist party) obtained 39 seats, up from 4, overtaking KESK as the third largest party. For a description of the parties participating to the election see supporting information file Parties S1.

## Results and Discussion

**Figure 1:**
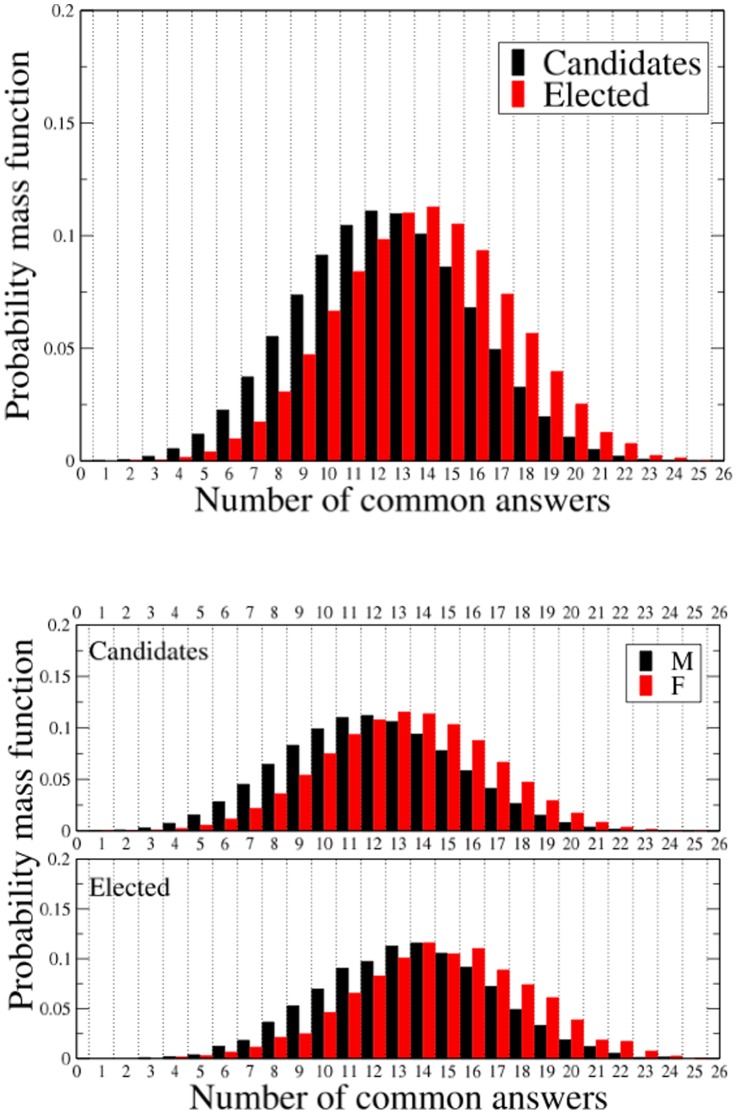
Probability mass functions of the number of common answers. Comparison between the probability mass function of the number of common answers between respondents, separately for candidates and elected (top panel) and disaggregated by gender (bottom panel). The probability mass functions are obtained by considering the answers of all 1,793 candidates who provided information about the gender, and the subset of 181 elected candidates.

We quantify the similarity of the political profile of two distinct candidates (or elected) by computing the number of common answers 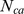 provided by them answering to the questionnaire. Fig. 1 shows the probability mass function 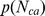 (calculated as fraction of participants) between respondents, separately reported for candidates and elected (Fig. 1a) and disaggregated by gender (Fig. 1b).

Fig. 1a shows that the similarity of political profile is higher for elected than for candidates. The information aggregation process of the voting therefore produces a convergence of the successful political profiles towards a consensus political profile present in the majority of voters. Fig. 1b shows the role of gender both for candidates and elected. We observe that female candidates and elected have political profiles more homogeneous than male candidates and elected respectively. We begin discussing the role of gender in shaping the political profile of candidates and elected.

### Political profile of candidates: the role of gender

Fig. 1b shows that considering altogether all female candidates and female elected they present a political profile more homogeneous than the corresponding ones of male candidates. Here we repeat the analysis taking into account the party membership of candidates and elected. In Table 1, we report the average number of common answers 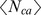 of male and female candidates calculated among respondents belonging to the same party. According to the table, the homogeneity of political profile of female candidates is higher than the one of male candidates in almost all parties with the only exception of SEN and KTP parties. The difference observed in the Table between the average political profile of male and female is statistically significant (at a 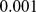 threshold) for many parties with a large number of candidates (SDP, PS, VAS, VIHR, RKP, KD, and SKP). The exceptions are KOK, KESK and PIR, where a t-test provides 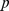-values larger the 0.1%. Such a statistically significant difference between political profile of male and female participants is also observed in the subset of elected participants for two parties. Specifically, we observe that elected females are more politically similar than elected males for SDP and PS, whereas elected females for KESK and VIHR present the opposite behavior. However, it should be noted that such a difference for these parties is statistically significant according to a t-test only for KESK.

**Table 1:** Summary statistics of the degree of similarity of political profiles of females and males, for both candidates and elected members, disaggregated by party.

	Number	NumberFemale	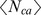 Female	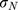 Female	NumberMale	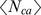 Male	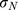 Male	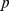 -value
**Candidates**								
Overall Sample	1,793	715	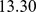	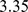	1,078	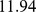	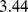	<0.001*
KOK	206	90	14.28	3.1	116	14.13	2.94	0.012
SDP	208	93	16.97	2.72	115	15.27	2.81	<0.001 *
PS	194	63	16.94	2.95	128	15.48	3.11	<0.001 *
KESK	200	84	15.37	2.96	115	15.31	3.08	0.322
VAS	191	83	16.72	2.81	106	16.18	2.8	<0.001 *
VIHR	212	110	15.38	3.09	102	14.79	2.77	<0.001 *
RKP	67	29	14.46	3.38	37	13.24	2.68	<0.001 *
KD	164	72	15.55	2.9	92	13.66	3.01	<0.001 *
PIR	82	10	12.04	2.65	71	11.34	2.92	0.109
SKP	94	34	17.67	2.68	60	16.38	2.93	<0.001 *
M2011	55	9	13.	2.45	46	12.45	2.83	0.251
IPU	40	9	13.69	2.88	31	12.67	3.24	0.066
VP	29	9	15.19	2.65	19	14.12	3.03	0.051
STP	19	4	14.5	2.51	15	13.65	2.72	0.455
SEN	18	6	12.47	2.29	12	14.26	2.98	0.032
KTP	8	1	NA	NA	7	15.14	2.89	NA
KA	10	7	14.29	2.76	3	11.67	2.08	0.131
**Elected**								
Overall Sample	181	78	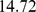	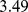	103	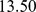	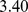	<0.001*
KOK	41	13	15.71	3.49	28	15.51	2.58	0.642
SDP	39	25	18.71	2.39	14	17.51	2.5	<0.001*
PS	35	10	18.31	2.48	25	16.27	3.08	<0.001*
KESK	28	11	16.64	2.56	17	18.4	2.5	<0.001*
VAS	14	6	19.	2.36	8	17.61	2.78	0.107
VIHR	10	5	14.7	3.13	5	17.2	3.49	0.109
RKP	8	5	19.	2.21	3	15.33	2.31	0.030
KD	6	3	19.33	1.15	3	18.67	1.15	0.519

The second column is the number of respondents in each group. 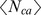 is the average number of common answers, 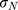 is its standard deviation, and the 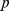-value is obtained through a t-test testing the null hypothesis that the values of 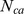 for males and females come from a distribution with the same mean. An asterisk indicates the cases when the 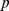-value is less than 0.001.

In summary, female candidates and elected candidates of some parties present an average political profile systematically more homogeneous than the corresponding male candidates or elected.

To verify whether our results are coherent with previous studies on the role of gender stereotypes in the interaction between candidates and voters we need to evaluate which aspects of the political profile are over-expressed in the political profile of female candidates and elected. To detect over-expressed political positions, we use a statistical approach recently proposed in ref. [12]. Consider a specific answer 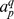 to question 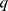, and indicate the actual number of people who give that answer as 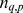. If we assume that the group 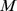 of male candidates and 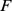 of female candidates are formed by randomly splitting the entire set of 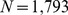 respondents in two groups of size 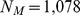 and 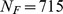, respectively, then the number of respondents 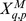 (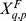) in group 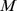 (group 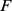) who give the answer 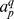 to question 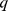 follows the hypergeometric distribution. Specifically, the probability 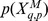 that 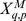 respondents from group 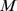 give the answer 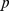 to the question 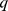 is
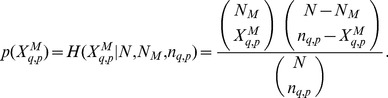
(1)


Similarly, for females, we have.
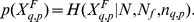
(2)


Equations (1) and (2) allow one to associate two different 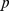-values with each observed number of respondents 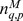 (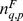) from group 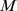 (group 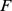) who give the answer 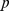 to question 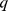. In particular we distinguish between a 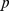-value of *over-expression*, which is calculated as the probability that 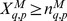 for the group 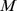, and as the probability that 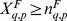 for the group 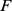, and a 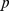-value of *under-expression*, calculated as the probability that 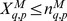 for the group 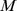, and that 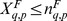 for the group 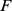. In Table 2 all the over-expressed and under-expressed answers of female and male candidates are reported, without conditioning on their party membership. In principle, the analysis could have been done separately for each party, in order to exactly remove any possible bias related to the different percentage of males and females in each party. However, unfortunately such an analysis cannot be performed, due to lack of sufficient statistics. In our analysis, we consider an answer to be over-expressed (OE) or under-expressed (UE) in a given group, either M or F, if the corresponding 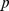-value is smaller than 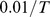, where 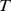 is the total number of tests, that is the total number of answers. This correction of the statistical threshold is used to properly correct the level of statistical significance for multiple hypothesis testing. Respondents are divided in only two groups, M and F. As a consequence, when few answers are possible, if an answer is over-expressed in one group then usually it is also under-expressed in the other group. We observe that in 13 out of 29 questions of the questionnaire, the groups of female and male candidates present a different endorsement to the answers. The topics of the political positions showing differences between the two genders concerns primarily welfare, social, educational and minority issues (Q1, Q2, Q4, Q5, Q22 and Q23), environmental issues (Q3 and Q26), taxes (Q9 and Q12), foreign affairs related to international aid and human rights (Q18 and Q20) and immigration policy (Q25). All the over expressed answer are coherent with the gender stereotypes reported in the political science literature that female candidates are kinder and more compassionate than men and caring more about social welfare issue [1], [2].

**Table 2:** Over-expressed (OE) and under-expressed (UE) endorsement of a specific answer (second column) given to the related question (first column) by candidates of different gender.

Question	Answer	F	M
Q1: Income inequality has increased in Finland. How should the situationbe approached?	A1. Income inequality should be reduced considerably.		UE
Q2: Should same sex couples also have the right to adopt childrenfrom outside the family?	A1: Yes.	OE	UE
	A2: No.	UE	OE
Q3: Should Fortum be granted a permit to replace two nuclear reactorsat Loviisa power plant?	A1: Yes.	UE	OE
	A2: No.	OE	UE
Q4: What should be done about the child benefit?	A2: It should be removed from high-income families.	UE	OE
	A4: Should not affect the amount of social assistance.	OE	UE
Q5: Should municipalities be obligated to offer the elderly guaranteedquality and availability of care?	A3: Yes.The elderly should have a right to good care	OE	UE
	A1. No.Responsibilities of municipalities shouldn’t increase.	UE	OE
Q9: Which cut of government expenditure would you choose first?	A1: Cut defense appropriations	OE	UE
	A2: Cut development appropriations	UE	OE
Q12: Which taxes would you primarily willing to increase?	A5: Energy taxes	OE	UE
	A9: No tax increases in general	UE	OE
Q18: Should Finland raise the issues of human rights anddemocracy more strongly in relations with Russia and China?	A1: Yes, Finnish foreign relations should be based oncitizens’ rights and generally accepted values.	OE	UE
	A2: Yes, but only as part of unifed EU relations.	UE	OE
Q20: What should be done about development aid?	A1: Finland should raise the level to 0.7%	OE	UE
	A4: The level should be lowered.	UE	OE
	A5: No money should be given in development aid.	UE	OE
Q22: What should the parliament do about the firearms law?	A2: Storing handguns at home should be banned.	OE	UE
	A4: The law should be relaxed.	UE	OE
Q23: Should learning the second national language be voluntary?	A1: Yes.	UE	OE
	A2: No.	OE	UE
Q25: How is the current immigration policyin your opinion?	A1: Too strict.	OE	UE
	A3: Too loose.	UE	OE
Q26: What should be done about protecting the Saimaa ringed seal?	A2: Current protection is sufficient.	UE	OE

The letter F indicates female candidates and M male candidates respectively.

One can object that the results obtained are affected by the heterogeneity of female candidates in different parties. In fact the highest percentage of female candidates (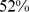 against an average percentage of 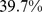) is observed for the VIHR party (The Green League, a centrist green liberal political party), which is more oriented towards a pro-environmental political profile. To test the role of this heterogeneity, we repeat our analysis removing from the investigated set of female candidates those parties that are presenting a percentage of female candidates that is too high or too low with respect to the average percentage observed (specifically, we remove female candidates from the VIHR (52% of female candidates), PIR (12%) and M2011 (16%) parties). For this subset of respondents, we again observe over-expression of 8 of the 13 questions reported in Table 2. These questions, and relative answers, are Q1, Q3, Q4, Q5, Q18, Q22, Q23 and Q26. The compassionate and welfare concerned profile of female candidates is confirmed also for the reduced subset.

### Political profile of elected respondents

We have shown in Fig. 1a that the degree of similarity of elected respondents is higher than the one of the whole set of candidates. We interpret this result as a manifestation of the process of information aggregation that is taking place in a voting procedure. In our view the voting procedure selects the political offers having major consensus in the population of voters or in large subsets of it.

To obtain evidence of information aggregation in the voting procedure, we compare the set of elected with the set on non-elected according to the answers provided to the Helsingin Sanomat survey, along the same lines followed in the characterization of female and male candidates. Table 3 summarizes the over-expressed and under-expressed answers for elected and non-elected respondents. The two groups show a statistically tested over-expression or under-expression of specific answers in 9 out of 29 questions. Over-expressions and under-expressions reported in Table 3 confirm the information aggregation process. In fact, elected parliament members are characterized by the lack of over-expression of more extreme political positions that were present in the survey. For instance, the set of elected presents under-expression of the political positions that Finland should never join the NATO, and should leave the European Monetary Union. Elected are characterized by a political offer with an over-expression of political positions saying that the current organization of the military service is fine, and that the age of retirement should neither be decreased nor increased by several years.

**Table 3:** Over-expressed (OE) and under-expressed (UE) endorsement of a specific answer (second column) given to the related question (first column) by non elected (NE) or by elected (E) candidates respectively.

Questions	Answers	E	NE
Q6: The age of retirement is currently 63–68. The lower limit should be…	A1: decreased.	UE	OE
	A4: increased by several years.	UE	OE
Q9: Which of the following proposals would you first choose to cut governmentexpenditure.	A4: Cut business subsidies.	OE	UE
Q10: Finland has taken part in financial support packages for certain euro countries incredit crisis. Which of the following best describes your view?	A6: Finland should leave the monetary unionas soon as possible.	UE	OE
Q16: How would you organize Finnish military service?	A2: The current system is good.	OE	UE
	A4: Move to voluntary service for both genders.	UE	OE
Q17: Should Finland apply for NATO membership?	A5: Never.	UE	OE
Q24: Is singing a religous song appropriate as part of the spring festival in schools?	A1: Yes, it is part of the tradition.	OE	UE
	A4: No, religious ceremonies do not belong to school festivals.	UE	OE
Q25: How is the current immigration policy in your opinion?	A1: Too strict.	UE	OE
Q27:Municipalities have outsourced services to private companies and third sectoroperators in recent years. Which of the following best describes your view?	A5: Outsourced services should be insourced again.Municipalities have a legal responsibility to offerservices, so they must also produce them.	UE	OE
Q29: What should be done about the proposed metropolitan municipality that Helsinkisupports, Espoo and Vantaa oppose?	A1: The current situation is good.	UE	OE

We observe that information aggregation is present both on the entire set of candidates and elected and within each party that has obtained a number of elected sufficient to allow us to perform a statistical validation. In fact, in Table 4 we report the average degree of similarity 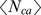 among members of the same party, for the two sets of elected and non-elected respondents. A t-test indicates that the average degree of similarity of elected members is significantly higher than the one of non-elected candidates in all the parties, with the only exception of VIHR, in which such a difference is also observed, but turns out not to be statistically significant at a 0.001 threshold.

**Table 4:** Summary statistics of the number of common answers 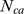 present in the political profile of elected and non-elected candidates, disaggregated by party.

	Number	NumberElected	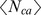 Elected	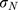 Elected	NumberNon-Elected	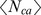 Non-Elected	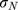 Non-Elected	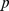 -value
Overall Sample	1,803	181	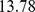	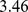	1,622	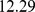	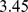	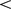 0.001*
KOK	206	41	15.46	2.95	165	13.77	3.01	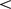 0.001*
SDP	208	39	17.97	2.46	169	15.57	2.83	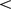 0.001*
PS	194	35	16.82	2.98	159	15.6	3.2	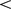 0.001*
KESK	200	28	17.64	2.55	172	14.91	3.05	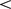 0.001*
VAS	191	14	17.97	2.68	177	16.33	2.83	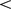 0.001*
VIHR	212	10	16.11	3.79	202	15.08	2.95	0.076
RKP	67	8	17.	3.36	59	13.45	2.87	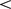 0.001*
KD	164	6	19.13	1.88	158	14.26	3.04	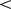 0.001*

The second column is the number of respondents in each group. 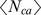 is the average number of common answers within members of each party, 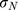 is its standard deviation, and the 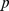-value is obtained through a t-test testing the null hypothesis that the the values of 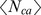 for elected and non-elected candidates come from a distribution with the same mean. An asterisk indicates a 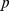-value less than 0.001.

The presence of a process of information aggregation is also supported by the analysis of correlation between the similarity of candidates’ profile and the number of votes they received. For each pair of respondents, we first calculate the similarity between their profile of answers and the geometric mean of the number of votes they received. We therefore calculate the Pearson correlation coefficient between these two quantities for all pairs of candidates of different groups of respondents. We first focus on the parties with elected candidates and we obtain the following values of intra-party correlation between similarity and votes: 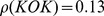, 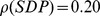, 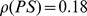, 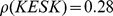, 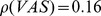, 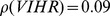, 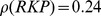, and 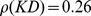. All these correlations are statistically significant with a statistical threshold of 0.001, and suggest that, within a party, pairs of candidates with higher similarity than average have been favored by the electorate. Similar results are obtained by using the arithmetic mean of votes in place of the geometric mean (see Table SI1 in Tables S1).

**Figure 2:**
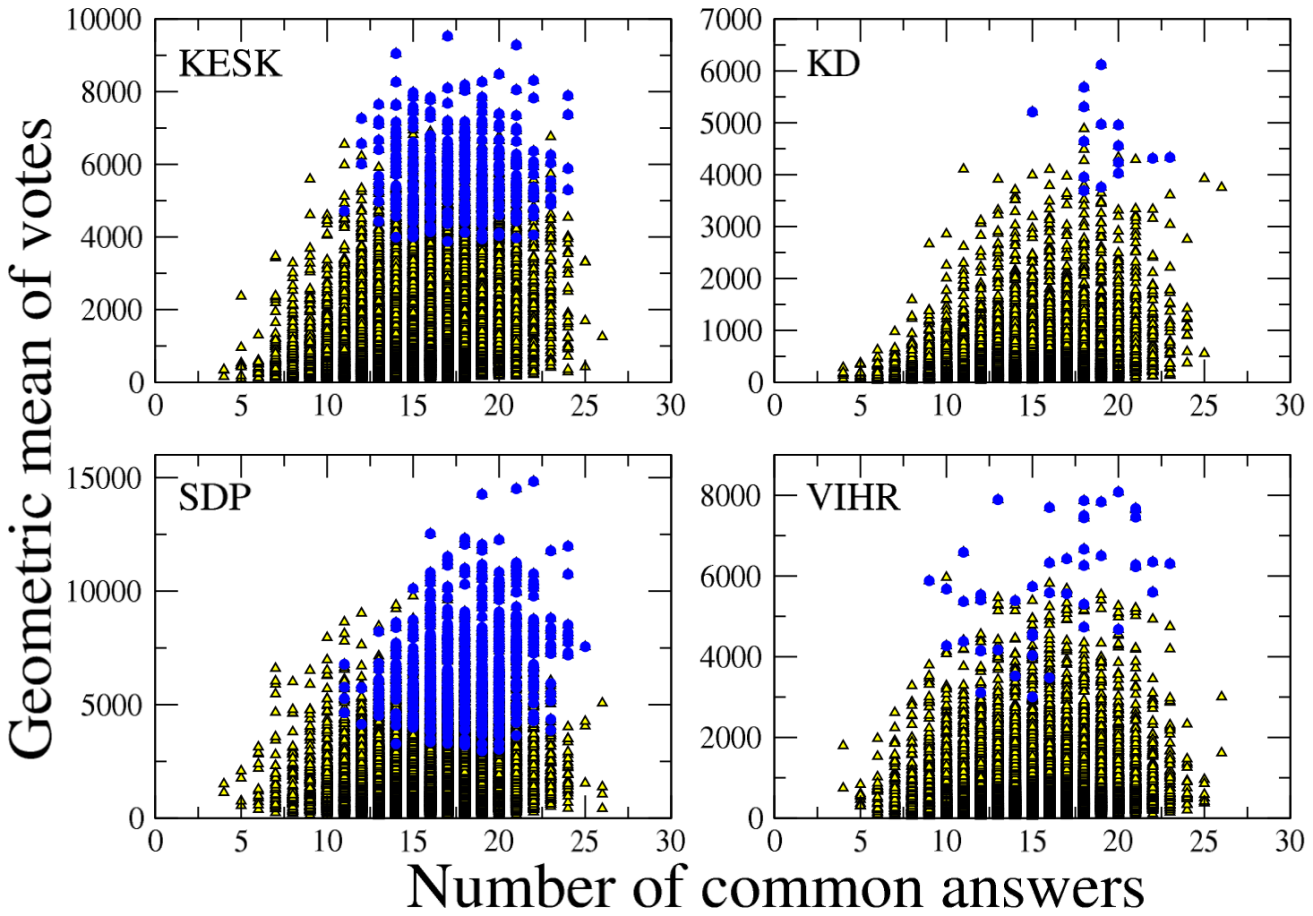
Scatter plot of votes VS number of common answers within each of four parties. Scatter plot of the geometric mean of the votes obtained versus the number of common answers for each pair of candidates belonging to the four parties KESK (top left), KD (top right), SDP (bottom left) and VIHR (bottom right). Yellow triangles denote pairs of non elected candidates, whereas blue circles denote pairs of candidates that have been elected.

In Fig. 2 we show the scatter plot of the geometric mean of the votes obtained versus the number of common answers for each pair of candidates belonging to the four parties KESK, KD, SDP and VIHR. For the three parties KESK, KD, and SDP the figure shows a general trend of higher geometric mean value of votes for higher values of the similarity 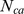. Elected of the KESK, KD and SDP parties have 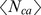 higher that non-elected candidates.

To test how information aggregation occurs among parties, we have compared the degree of similarity among members of different parties for the two sets of elected and non elected candidates. Though the similarity among members of different parties is, on average, lower than the similarity among members of the same party, the similarity among different parties is, in several cases, higher for elected than for non-elected participants. In fact, the results, reported in Table 5, show that the voting process has two major effects: (i) it tends to smooth out the differences between several parties competing for the political center or for contiguous political areas, and (ii) it increases political polarization when the successful candidates bear political profiles which are at the boundary of some political dimension. In Table 5 we observe the effect (i), i. e., higher values of similarity for elected than non-elected, for several pairs of parties at a 0.001 statistical threshold. Exceptions are the pairs PS-VIHR and KESK-VAS which are showing lower similarity in elected rather than in non-elected at a 0.001 threshold, therefore supporting the presence of the effect stated in (ii).

**Table 5:** Summary statistics of the inter-party average number of common answers 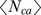 present in the political profile of elected (E) and non-elected (NE) candidates, disaggregated by pairs of parties.

Party 1(P1)	Numberof E in P1	Numberof NE in P1	Party 2(P2)	Numberof E in P2	Numberof NE in P2	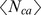 E	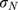 E	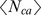 NE	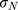 NE	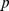 -value
KOK	41	165	SDP	39	169	12.17	2.56	11.67	3.11	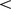 0.001*
KOK	41	165	PS	35	159	11.98	2.91	11.19	2.89	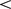 0.001*
KOK	41	165	KESK	28	172	14.47	2.66	13.21	3.08	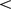 0.001*
KOK	41	165	VAS	14	177	9.76	2.48	9.89	3.1	0.223
KOK	41	165	VIHR	10	202	11.4	2.67	10.55	3.09	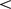 0.001*
KOK	41	165	RKP	8	59	14.73	2.68	12.5	3.06	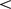 0.001*
KOK	41	165	KD	6	158	13.41	2.79	12.12	3.09	<0.001*
SDP	39	169	PS	35	159	13.26	2.68	12.95	3.11	<0.001*
SDP	39	169	KESK	28	172	14.08	2.83	13.57	3.16	<0.001*
SDP	39	169	VAS	14	177	16.24	2.61	14.93	2.87	<0.001*
SDP	39	169	VIHR	10	202	14.01	3.03	13.64	3.02	0.017
SDP	39	169	RKP	8	59	13.31	3.21	12.21	2.92	<0.001*
SDP	39	169	KD	6	158	16.45	2.52	13.47	3.03	<0.001*
PS	35	159	KESK	28	172	14.27	2.74	12.77	3.08	<0.001*
PS	35	159	VAS	14	177	12.48	2.84	12.3	3.13	0.225
PS	35	159	VIHR	10	202	9.36	2.68	10.35	2.94	<0.001*
PS	35	159	RKP	8	59	11.13	2.61	9.75	2.79	<0.001*
PS	35	159	KD	6	158	15.34	2.72	13.51	2.94	<0.001*
KESK	28	172	VAS	14	177	11.77	2.96	12.43	3.2	<0.001*
KESK	28	172	VIHR	10	202	12.16	3.07	12.26	3.16	0.588
KESK	28	172	RKP	8	59	15.53	2.97	12.85	3.07	<0.001*
KESK	28	172	KD	6	158	16.58	2.21	13.8	3.03	<0.001*
VAS	14	177	VIHR	10	202	14.29	3.06	14.44	3.03	0.565
VAS	14	177	RKP	8	59	11.85	2.99	11.51	2.87	0.219
VAS	14	177	KD	6	158	14.61	2.43	12.68	3.17	<0.001*
VIHR	10	202	RKP	8	59	13.36	3.39	12.35	2.94	0.002
VIHR	10	202	KD	6	158	13.37	2.52	11.53	3.01	<0.001*
RKP	8	59	KD	6	158	15.6	3.38	11.63	3.08	<0.001*

The second (third) column is the number of elected (non elected) respondents in the first party (P1), while the fifth (sixth) column is the number of elected (non elected) respondents in the second party (P2). 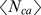 is the average number of common answers between the members of the two parties, 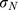 is its standard deviation, and the 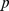-value is obtained through a t-test testing the null hypothesis that the the values of 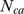 for elected and non-elected candidates come from a distribution with the same mean. An asterisk indicates a 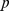-value less than 0.001.

By repeating the analysis investigating the relationship between the geometric mean of votes and the similarity 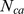 for pairs of candidates and elected belonging to different parties, we obtain the results reported in Table 6. The results confirm that, on average, inter-party correlation between similarity and number of votes is positive for parties competing for the political center (for example KESK, KD, and SDP) or for contiguous areas of political consensus (for example SDP and VIHR or PS and KESK). Again a different behavior is observed when parties are proposing opposite views about main political problems, as in the case of the pairs KOK-VAS (The Left Alliance, founded on earlier left-wing parties), KESK-VAS or PS-VIHR. In these cases pairs of candidates of opposite parties with political profiles of lower similarities have been selected during the voting process. The corresponding results obtained by using the arithmetic mean instead of the geometric mean are shown in Table SI2 in Tables S1.

**Table 6:** Correlation between the number of common answers 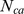 and the geometric mean of votes of pairs of candidates belonging to different parties.

Party 1	Party 2	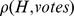
KESK	KD	0.213*
RKP	KD	0.184*
KESK	RKP	0.179*
PS	KD	0.172*
SDP	KD	0.17*
KOK	RKP	0.167*
PS	KESK	0.16*
VAS	KD	0.107*
SDP	VAS	0.103*
PS	RKP	0.103*
KOK	KESK	0.079*
SDP	RKP	0.074*
VIHR	KD	0.071*
VIHR	RKP	0.064*
VAS	RKP	0.058*
VAS	VIHR	0.044*
SDP	VIHR	0.039*
KOK	KD	0.037*
SDP	KESK	0.025*
KOK	PS	0.01
KOK	VIHR	0.001
SDP	PS	−0.001
KOK	SDP	−0.015
PS	VAS	−0.034*
KESK	VAS	−0.046*
KESK	VIHR	−0.047*
KOK	VAS	−0.051*
PS	VIHR	−0.115*

An asterisk indicates statistically significant correlation at the 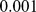 threshold. Pairs of parties are sorted in decreasing order of correlation.

**Figure 3:**
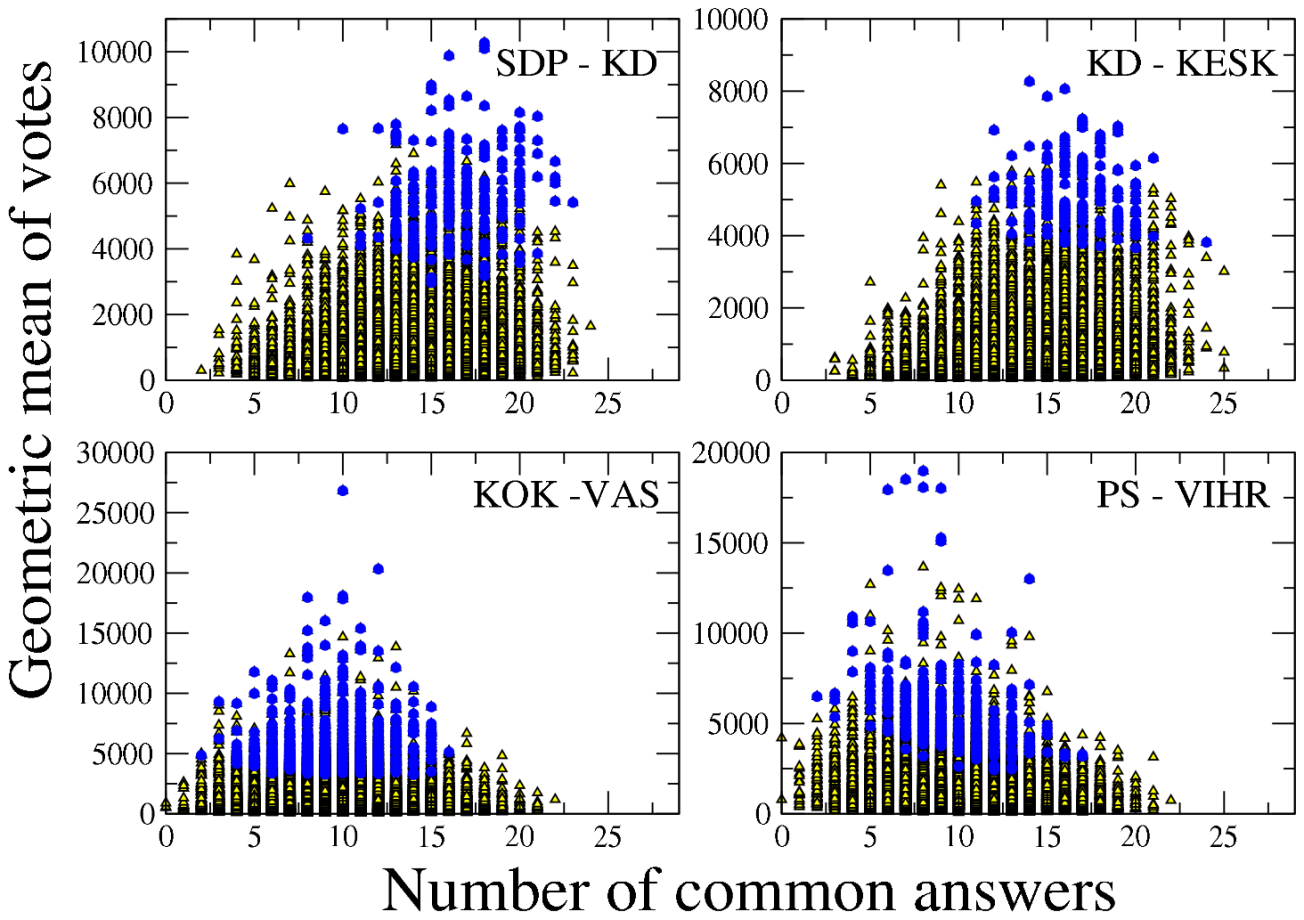
Scatter plot of votes VS number of common answers for candidates belonging to different parties. Scatter plot of the geometric mean of the votes obtained versus the number of common answers for pairs of candidates belonging to four pairs of parties. Each pair of candidates is formed by one candidate from one party and the other from a different party. The pairs of parties are: SPD-KD (top left), KD-KESK (top right), KOK-VAS (bottom left) and PS-VIHR (bottom right). Yellow triangles denote pairs of non elected candidates whereas blue circles denote pairs of candidates that have been elected.

In Fig. 3 we show the scatter plot of the geometric mean of the votes versus the number of common answers 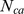 for each pair of candidates belonging to different parties. Specifically, we show the pairs of parties: SDP-KD (top left), KD-KESK (top right), KOK-VAS (bottom left) and PS-VIHR (bottom right). The top panels of the figure show parties which are competing for the center of the political scene whereas the bottom panels show pairs of parties that offer opposite political profiles. The top panels and the bottom panels show a quite different behavior. The top panels are similar to what is observed inside a single party (see Fig. 2) confirming that candidates of these parties are all competing to offer a political profile tailored on a voter of the center of the political various political dimensions. The bottom panels shows that, on average, most successful pairs of candidates present distinct political offers, which are more distant than average offers of the pairs of candidates belonging to the two parties. Specifically, the bottom panels show a market oriented party (KOK) against a party with roots on the communist ideas of state intervention and state planning (VAS) in the left panel and, a nationalist and populist party (PS) against a party broadly supporting civil rights and considering aspects like environmental compatibility, sustainable growth and international solidarity (VIHR) in the right panel.

### Conclusions

In summary we have quantitatively verified that (i) on average female candidates are publicly offering political profiles which are more compassionate and caring more about social welfare issues than male candidates and (ii) the voting procedure acts as a process of information aggregation. It should be noted that our results present a new research perspective on the role of gender stereotypes in voting. In fact in our study we quantitatively detect the presence of gender stereotypes in the political offer of female candidates. The political science literature has been mainly focused so far on the topic of the role of gender as information conveying compassionate and welfare oriented traits of the female candidates to the low-informed voter [1]–[4]. In our study we reverse the perspective and we show that female candidates are effectively signaling compassionate and welfare oriented political traits. Our study also shows that elected parliament members are characterized by the lack of over-expression of more extreme political positions supporting the view that voting is an information aggregation procedure. We also show that this information aggregation can occur in at least two distinct ways one reflecting competition among candidates for providing an offer satisfying the political center of the nation and/or of each party and the other involving offers of distinctive political profiles of candidates of political parties bearing extreme positions in some political dimensions.

## Supporting Information

Tables S1 **Table SI1**, Intra-party correlation between mean number of votes of pairs and similarity, but with arithmetic mean of votes in place of geometric mean. All the correlation values are statistically significant with a statistical threshold of 0.001. **Table SI2**, Like Table VI of the main text, but with arithmetic mean of votes in place of geometric mean.
(PDF)

Parties S1 **Basic information about Finnish political parties.**
(PDF)

Questions S1 **English translation of the questionnaire used to perform the survey.**
(PDF)
