# Supplementary material for: Quantitative Analysis of Gender Stereotypes and Information Aggregation in a National Election
Source: PLoS One. 2013 Mar 26;8(3):e58910. doi: 10.1371/journal.pone.0058910 (PMC3608643; doi:10.1371/journal.pone.0058910)
Supplement: Tables S1 — Table SI1, Intra-party correlation between mean number of votes of pairs and similarity, but with arithmetic mean of votes in place of geometric mean. All the correlation values are statistically significant with a statistical threshold of 0.001. Table SI2, Like Table VI of the main text, but with arithmetic mean of votes in place of geometric mean. (PDF) [file pone.0058910.s001.pdf]

TABLE I: Intra-party correlation between mean number of votes of pairs and similarity, but with arithmetic mean of votes in place of geometric mean. All the correlation values are statistically significant with a statistical threshold of 0.001.

Party	$\rho(H, votes)$
KOK	0.10
SDP	0.18
PS	0.12
KESK	0.25
VAS	0.13
VIHR	0.04
RKP	0.23
KD	0.22

TABLE II: Like Table VI of the main text, but with arithmetic mean of votes in place of geometric mean.

Party 1	Party 2	$\rho(H, votes)$
KESK	KD	0.187*
RKP	KD	0.185*
KESK	RKP	0.172*
KOK	RKP	0.156*
PS	KESK	0.107*
SDP	KD	0.093*
SDP	RKP	0.088*
SDP	VAS	0.088*
PS	KD	0.086*
PS	RKP	0.078*
VAS	RKP	0.065*
VAS	KD	0.061*
SDP	VIHR	0.058*
VIHR	RKP	0.057*
KOK	KESK	0.04*
VIHR	KD	0.028*
SDP	KESK	0.017
VAS	VIHR	0.01
KOK	VIHR	0.009
KOK	PS	-0.008
KOK	SDP	-0.009
KOK	KD	-0.017
SDP	PS	-0.028*
KOK	VAS	-0.04*
KESK	VAS	-0.047*
PS	VAS	-0.049*
KESK	VIHR	-0.052*
PS	VIHR	-0.079*
