# Supplementary material for: Quantitative Analysis of Gender Stereotypes and Information Aggregation in a National Election
Source: PLoS One. 2013 Mar 26;8(3):e58910. doi: 10.1371/journal.pone.0058910 (PMC3608643; doi:10.1371/journal.pone.0058910)
Supplement: Parties S1 — Basic information about Finnish political parties. (PDF) [file pone.0058910.s002.pdf]

Party	Name	Founded	European Parliamentary Group	Votes in 2011	Votes in 2007	Members
KOK	National Coalition Party	1918 [1]	Group of European People's Party	20.4%	22.3%	41,000 [2]
SDP	Social Democratic Party	1899 [1]	Progressive Alliance of Socialists and Democrats	19.1%	21.4%	50,000 [2]
PS	True Finns	1995 [3]	Europe of Freedom and Democracy	19.1%	4.1%	5,000 [2]
KESK	Centre Party	1906 [1]	Alliance of Liberals and Democrats for Europe	15.8%	24.1%	163,000 [2]
VAS	Left Alliance	1990 [1]	European United Left-Nordic Green Left [1]	8.1%	8.8%	9,100 [2]
VIHR	The Green League	1988 [1]	The Greens-European Free Alliance	7.3%	8.5%	4,600 [2]
RKP	Swedish People's Party	1906 [1]	Alliance of Liberals and Democrants for Europe	4.3%	4.6%	28,000 [2]
KD	Christian Democrats	1958 [4]	Group of European People's Party	4.0%	4.9%	13,000 [2]
PIR	The Pirate Party	2008 [5]		0.5%	-	3,700 [5]
SKP	Communist Party of Finland	1994 [6]		0.3%	0.7%	2,500 [6]
M2011	Change 2011	2009 [7]		0.3%	-	
IPU	Independence Party	1994 [3]		0.1%	0.2%	
VP	Freedom Party	2009 [8]		0.1%	-	
STP	Workers' Party of Finland	1999 [3]		0.1%	0.1%	
SEN	Senior Party of Finland	1991 [3]		0.1%	0.6%	
KTP	Communist Workers' Party	1989 [3]		0.1%	0.1%	
KA	For the Poor	2002 [3]		0.0%	0.1%	

Notes

[1]In the 2004-2009 European Parliament. They have no seats in the 2009-2014 parliament.

References

[1] P. Saukkonen, Suomen poliittinen järjestelmä (2008).

[2] Kauppalehti, Perussuomalaisilla hurja tahti: ”Jäseniä tulee ovista ja ikkunoista”, (2011). Retrieved 18 Jun. 2012 http://www.kauppalehti.fi/5/i/talous/uutiset/etusivu/uutinen.jsp?oid=20110364985

[3] Opetusministeriö, Puoluerekisteriin merkityt ja siit poistetut puolueet (2011).

[4] Kristillisdemokraatit, Pḧkinänkuoressa. Retrieved 19 Jun. 2012 http://www.kristillisdemokraatit.fi/KD/www/fi/politiikka/Historia/pahkinankuoressa.php

[5] Piraattipuolue, Historia ja tilastoja. Retrieved 18 Jun. 2012 http://www.piraattipuolue.fi/puolue/historia

[6] Suomen Kommunistinen Puolue, Tietoa SKP:stä. Retreived 18 Jun. 2012 http://www.skp.fi/puolue/tietoa-skp-sta/

[7] Muutos 2011, Mikä Muutos? Retrieved 18 Jun. 2012 http://www.muutos2011.fi/mika-muutos/

[8] Vapauspuolue, Vapauspuolueen historia lyhyesti. Retrieved 18 Jun. 2012 http://www.vapauspuolue.fi/sivut/?option=com_content&view=article&id=79
