# Supplementary material for: Quantitative Analysis of Gender Stereotypes and Information Aggregation in a National Election
Source: PLoS One. 2013 Mar 26;8(3):e58910. doi: 10.1371/journal.pone.0058910 (PMC3608643; doi:10.1371/journal.pone.0058910)
Supplement: Questions S1 — English translation of the questionnaire used to perform the survey. (PDF) [file pone.0058910.s003.pdf]

Questions

Q1. Income inequality has increased in Finland since the mid-1990s. How should the situation be approached?

A1. Income inequality should be reduced considerably.

A2. Income inequality should be reduced slightly.

A3. Income inequality is currently at an appropriate level.

A4. Income inequality should be allowed to increase with restraint.

A5. Income inequality should be allowed to increase freely.

Q2. In 2009 the parliament accepted a law making it possible for registered same-sex couples to adopt step children. Should same sex couples also have the right to adopt children from outside the family?

A1. Yes.

A2. No.

Q3. In the spring 2010 the government granted two permits for nuclear power plants. The third applicant, Fortum, was for granted a license, but hopes to be granted a permit to replace the two reactors at Loviisa power plant. Should Fortum be granted the permit?

A1. Yes.

A2. No.

Q4. Child benefit is paid for each child under 17 living in Finland, regardless of the parents' income. What should be done about the child benefit?

A1. Child benefit should be removed from high-income families.

A2. Child benefit should be made progressive according to the parents' income.

A3. The current situation should be maintained.

A4. Child benefit should not influence the amount of social assistance, but those living on social assistance should receive both that and child support.

Q5. The Ministry of Social Affairs and Health is preparing a law on the elderly that may include a guarantee of the quality and availability of care. Should this guarantee be included and municipalities be obligated to offer a subjective right to this care?

A1. No. The responsibilities of municipalities should not be increased.

A2. No. A recommendation for the level or care is sufficient.

A3. Yes. The elderly should have a subjective right to good care.

Pensions

Q6. The age of retirement is currently 63-68. The lower limit should be...

- A1. decreased.
- A2. kept as is.
- A3. increased by one year.
- A4. increased by several years.

Q7. The average age of retirement is below 60 years. The main reason for this is disability pensions. In addition to coping and well-being at work, other solutions to increasing the average retirement age have been proposed. Which of the following would you principally use to extend careers?

- A1. Remove the possibility for part-time pension.
- A2. Remove the right to extended unemployment allowance before retirement.
- A3. Increase the lower limit of old-age pension above 63 years.
- A4. All of the above.
- A5. None of the above.

Q8. Pensions are increased according to an index that weights consumer prices by 80% and wage development by 20%. Since mid-1990s when the index was introduced, pensions have lagged general income development. What should be done about the index?

- A1. Wage development should have more weight on the index than prices.
- A2. The index should be change back to weighting both by 50%.
- A3. The weight of wage development should be increased slightly.
- A4. There is no need to change the current index.
- A5. The weight of consumer prices should be higher.

The Economy

Q9. The government after the election may decide to cut government expenditure. The following proposals have been mentioned during the elections. Which would you choose first?

- A1. Cut defense appropriations.
- A2. Cut development appropriations.
- A3. Cut agricultural subsidies.
- A4. Cut business subsidies.

A5. Remove child benefit from high-income families.

A6. Untie social benefits from economic indices.

A7. Cut state aid to municipalities.

A8. Remove the subjective right for day-care.

A9. Something else.

A10. There is no need to cut government expenditure.

Q10. Finland has among other euro countries taken part in rescue packages worth hundreds of billions aimed at helping countries in debt crises. In the spring 2010 Finland committed to lending Greece 1.6 billion euro. Additionally Finland pledged 8 billion in collateral on the 750 billion euro European Financial Stability Facility. Finland may yet have to pledge more collateral. Which of the following best describes your view?

A1. Finland should support crisis countries due to European solidarity.

A2. Supporting crisis countries is in Finland's best interests, as the default of one country would endanger the whole euro system and the Finnish economy.

A3. Greece should have been left to enter bankruptcy proceedings to avoid the danger of moral hazard.

A4. Supporting Greece and Ireland was a mistake, as each country should take care of their own business.

A5. Joining the European Monetary Union was a mistake and we are paying for that now.

A6. Finland should leave the monetary union as soon as possible.

Q11. The financial crisis has spurred discussion of the financial sector's taxation and part in paying the costs of the crisis. The European Commission has proposed a global transaction fee that would apply to trades with bonds, shares, currencies and derivatives. Which of the following best describes your view?

A1. The transaction tax would hinder borrowing by people and companies, inhibiting growth. Under no circumstances should it be introduced.

A2. The transaction tax should be introduced if it was introduced globally.

A3. The EU should introduce the transaction tax even if the rest of the world would not participate.

Taxes

Q12. Which taxes would you primarily be willing to increase? (Choose one.)

A1. The income tax.
A2. The capital gains tax.
A3. The corporate tax.
A4. The value-added tax.
A5. Energy taxes.
A6. The property tax.
A7. I would reintroduce the wealth tax.
A8. None of the above.
A9. No tax increases in general.

Q13. The government uses taxation not only to fund expenses, but also as to further societal goals. Tax exemptions can be used to guide citizens' decisions. In the year 2009 these exemptions cost 13 billion euro in lost tax revenue. This is equal to approximately one quarter of the government budget. Revenue lost due to exemptions must be raised by increasing other taxes. Which of the following would you be willing to cut first? (Choose one.)

A1. The exemption of home-sales from the capital gains tax (lost revenue: 900 million in 2009).
A2. The exemption of child benefits (lost revenue: 510 million in 2009).
A3. The exemption of housing benefits (lost revenue: 280 million in 2009).
A4. The exemptions of employee benefits (lost revenue: 260 million in 2009).
A5. The tax credit for domestic expenses (lost revenue: 245 million in 2009).
A6. The exemption of union dues (lost revenue: 210 million in 2009).
A7. None of the above.

Q14. What should be done about the deductibility of mortgage interest?

A1. Removed completely.
A2. Removed gradually with a long transition period.
A3. Kept as is.
A4. It should be expanded.

Q15. Property tax is paid on both developed and undeveloped lots. Should a property tax also be introduced for forests and fields?

A1. Yes.
A2. No.

Defense

Q16. Of the European countries only Greece, Cyprus and Finland have compulsory military service for all men. How would you organize Finnish military service?

A1. Women should also have compulsory military service.

A2. The current system is good.

A3. We should move to more selective conscription.

A4. We should move to voluntary service for both genders.

A5. We should remove military service.

Q17. Should Finland apply for NATO membership?

A1. Yes, already during the next parliamentary term.

A2. Yes, but not yet during the next parliamentary term.

A3. Yes, if Sweden also applies.

A4. Not during the next parliamentary term.

A5. Never.

Foreign Affairs

Q18. Should Finland raise the issues of human rights and democracy more strongly in relations with Russia and China?

A1. Yes, Finnish foreign relations should be based on citizens' rights and generally accepted values.

A2. Yes, but only as part of unified EU relations.

A3. No, financial interests are more important.

A4. No, the treatment of Russian and Chinese citizens is none of our business.

Q19. Early this year Russia prohibited foreign land owning near their borders. Russians have been able to purchase land in Finland nearly without limits. What should be done about it?

A1. Finland should generally restrict foreign ownership of property and land.

A2. Finland should restrict Russian ownership of property and land until there is reciprocity.

A3. Foreign rights to ownership of property and land should not be changed.

Q20. Finland is committed to a UN target of 0.7% of GDP for appropriations for development cooperation. Last year Finland gave 965.6 million euro, which is 0.55%, for development cooperation. What should be done about development aid?

A1. Finland should raise the level to 0.7% according to the commitment.
A2. The level can be raised moderately if the government budget allows.
A3. The level should not be raised.
A4. The level should be lowered.
A5. No government money should be given in development aid.

Q21. If Finland was on Facebook, which 3 countries should Finland first request as friends?

A1. Sweden
A2. Norway
A3. Denmark
A4. Estonia
A5. Iceland
A6. Russia
A7. China
A8. India
A9. The USA
A10. Canada
A11. Britain
A12. Germany
A13. France
A14. Spain
A15. Italy
A16. Poland
A17. Turkey
A18. Iran
A19. Israel
A20. Syria
A21. Tunisia
A22. Ghana
A23. Tanzania
A24. Brazil
A25. Bolivia
A26. Cuba

Domestic Affairs

Q22. The firearms law was tightened in 2010 by e.g. raising the age limit for handgun permits to 20 years. What should the new parliament do about the firearms law?

A1. Handguns should be banned completely.

A2. Storing handguns at home should be banned.

A3. The law is good as it is now.

A4. The law should be relaxed.

Q23. In comprehensive school it is compulsory to learn two foreign languages, one of which must be the second national language [Swedish or Finnish]. Should learning the second national language be voluntary?

A1. Yes.

A2. No.

Q24. Is singing the Suvivirsi [a traditional religious song] appropriate as part of the spring festival in schools?

A1. Yes, it is part of the tradition.

A2. Yes, as long as atheists and members of other religions are given a chance not to participate in signing.

A3. Yes, as long as atheists and members of other religions are given a alternative activity.

A4. No, religious ceremonies do not belong to school festivals.

Q25. During the 2007-2011 parliamentary term various decisions were made to tighten Finnish immigration policy. How is the current immigration policy in your opinion?

A1. Too strict.

A2. Fine.

A3. Too loose.

Q26. Saimaa ringed seal is classified an endangered species. The population is currently approximately 270 individuals and it is thought according to some estimates to be ”extremely likely” to become extinct. It has been recently protected mainly through voluntary action, for example fishing restrictions and a voluntary net fishing ban in the early summer. What should be done about protecting the Saimaa ringed seal?

A1. The protection is already too intense.

A2. Current protection is sufficient.
A3. Net fishing should be completely banned in central nest areas.
A4. A ban on net fishing should extended to the whole Lake Saimaa.
A5. In addition to a ban on net fishing, permit requirements for coastal construction should be tightened.

Municipalities

Q27. Municipalities have outsourced services to private companies and third sector operators in recent years. Pressure to outsource services is still increasing. Which of the following best describes your view?

A1. All municipal services should be opened for competition and municipalities should automatically use competitive bidding for all services.
A2. Outsourcing should be increased considerably, because companies can produce services more efficiently, but municipalities could still decide when to use competitive bidding.
A3. Outsourcing should be increased, but municipalities should learn to better employ competitive bidding to improve both price and quality of services.
A4. No more services should be outsourced, since outsourcing endangers the quality of public services and does not result in significant savings.
A5. Outsourced services should be insourced again. Municipalities have a legal responsibility to offer services, so they must also produce them.
A6. None of the above.

Q28. Due to municipal mergers the number of municipalities has decreased to 336. Mergers are used to seek higher efficiency in municipal management, because many small municipalities have trouble coping with their legal responsibilities regarding the production of services. What would be a suitable number of municipalities?

A1. Under 170
A2. 170-229
A3. 230-289
A4. 290-339
A5. 340-400
A6. Over 400

Q29. There has been a long debate about merging the capital region cities. Supporters think a merger would benefit especially zoning and traffic planning. The leaders of Helsinki support a metropolitan municipality, while Espoo and Vantaa oppose it. The next parliament and government also have influence. What should be done?

A1. The current situation is good.

A2. Cooperation must be increased, but voluntarily on the municipalities' initiative.

A3. The government should create a metropolitan municipality, even by force.

A4. The municipal borders should be maintained, but a new elected regional council should be created that would take on a large part of the municipalities' current responsibilities.

Q30. The state balances municipal tax revenue by transferring money from richer municipalities to poorer ones. The system guarantees each municipality a tax revenue level of 91.86% the average. The largest payers of the system are Helsinki and Espoo, from where approximately 500 million euro is transferred to poorer municipalities this year. What should be done about the system?

A1. More tax revenue should be transferred from the capital region to the rest of Finland.

A2. The current balancing system is good.

A3. The capital region should get to keep a larger share of their tax revenue.

A4. The system should be abolished.

Government

Q31. Choose three parties that should at least be in the next government. Choose exactly three. (Parties listed in order of registration.)

A1. Sdp

A2. Keskusta

A3. Kokoomus

A4. Rkp

A5. Kristillisdemokraatit

A6. Vihret

A7. Perussuomalaiset

A8. Skp

A9. Ktp

A10. Stp

A11. Itsenisyyspuolue

A12. Kyhien asialla

A13. Piraattipuolue

A14. Muutos 2011

A15. Vapauspuolue
